# 95. Reduced Antibiotic Duration Defaults in Outpatient Automated Dispensing Cabinets Change Antibiotic Prescribing Habits in a Tertiary VA Healthcare System

**DOI:** 10.1093/ofid/ofab466.297

**Published:** 2021-12-04

**Authors:** Nicholas J Newman, Usha Stiefel, Robert C Wenzell, Daniel Papell, Jeffrey Cooney, Sharanie Sims, Amy H Shumaker, Ukwen Akpoji

**Affiliations:** 1 VA Northeast Ohio Healthcare System, Beachwood, Ohio; 2 Cleveland VA Medical Center, Cleveland, OH; 3 Northeast Ohio Veterans Affairs Healthcare System, Cleveland, Ohio; 4 Louis Stokes Cleveland VA Medical Center, Cleveland, OH

## Abstract

**Background:**

Ten percent of adult, outpatient visits result in an antibiotic prescription (Rx). At the start of our intervention, our VA healthcare system consisted of 13 community-based outpatient clinics (CBOCs), 9 of which did not have an onsite pharmacy but utilized automated dispensing cabinets (ADCs) for prepackaged outpatient Rxs. ADC antibiotic orders are generated from electronic medical record (EMR) order sets. The stewardship team shortened the durations of 5 antibiotics in the ADC order sets to make them consistent with current literature and guidelines. We assessed the impact of these changes on antibiotic prescribing habits.

**Methods:**

We compared outpatient antibiotic Rx data between 10/1/2018-9/30/2019 (pre-intervention) and 10/1/19-9/30/20 (post-intervention) from 8 CBOCs with ADCs (1 closed during the pandemic). Amoxicillin-clavulanate 875/125mg (AMC), cephalexin 500mg (CPH), levofloxacin 500mg and 750mg (LEV 500 and LEV 750), and sulfamethoxazole-trimethoprim 800/160mg (SXT) prescription durations were all reduced by 3 days. Process metrics included days supplied/1000 prescriptions (DS/1000 Rx), median DS, and ADC utilization rates. We used Mann-Whitney U and correlation statistical analyses to assess differences and associations.

**Results:**

The DS/1000 Rx of antibiotics with a default duration change decreased in the post-intervention phase for CBOCs with ADCs (AMC, -25.4%; CPH, -21.1%; LEV 500, -18.9%; LEV 750, -28.0%; SXT, -27.4%). The median DS for these antibiotics all reduced by 3 days in concordance with new ADC prescriptions defaults (AMC, 10 vs 7 days, P< 0.001; CPH, 10 vs 7 days, P< 0.001; LEV 500, 8 vs 5 days, P< 0.001; LEV 750, 8 vs 5 days, P< 0.001; SXT 10 vs 7 days, P< 0.001). Due to COVID-19, 7/8 ADC CBOCs closed for in-person visits from 3/20/20-5/4/20. ADC utilization was inversely proportional to DS/1000 Rx for most antibiotics (R: -0.51 to -0.77) except SXT.

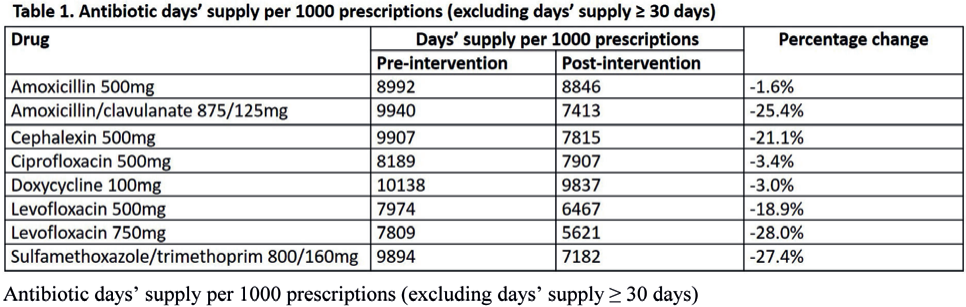

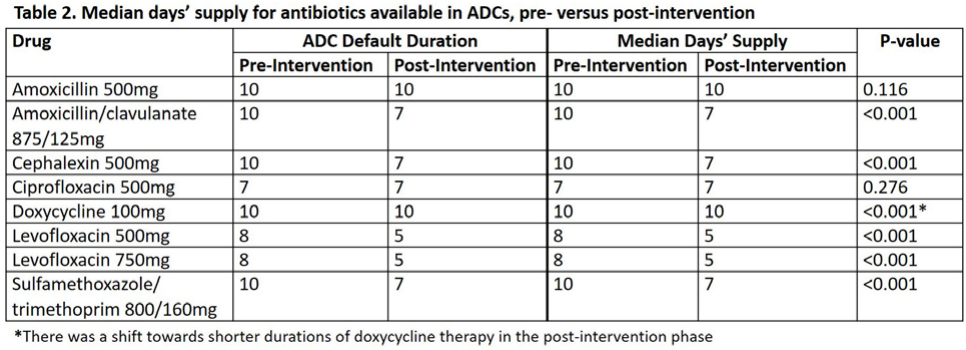

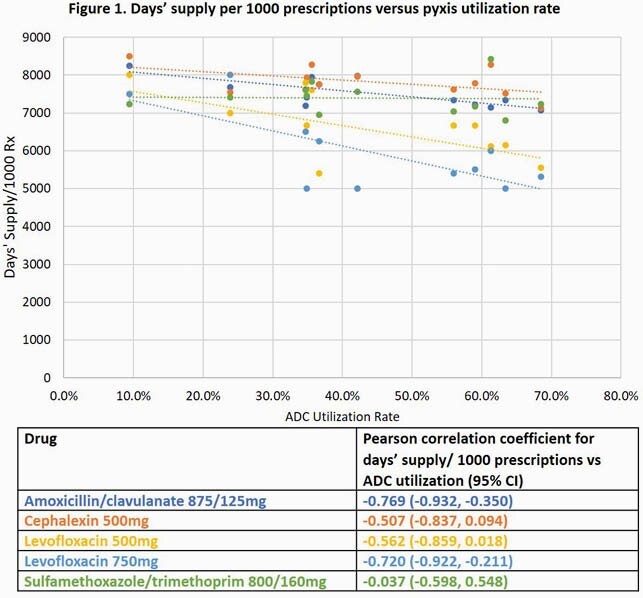

**Conclusion:**

EMR-driven reductions in ADC default Rx durations led to a corresponding decrease in overall outpatient antibiotic prescribing. Higher DS/1000 Rx were often associated with lower ADC utilization. Informatics-driven antibiotic interventions may be potential outpatient stewardship tools to increase guideline-concordant prescribing across multisite healthcare systems.

**Disclosures:**

**Sharanie Sims, PharmD**, **AbbVie (formerly Allergan**) (Speaker’s Bureau)

